# Iota-Carrageenan Inhibits Replication of SARS-CoV-2 and the Respective Variants of Concern Alpha, Beta, Gamma and Delta

**DOI:** 10.3390/ijms222413202

**Published:** 2021-12-08

**Authors:** Maria Fröba, Maximilian Große, Christian Setz, Pia Rauch, Janina Auth, Lucas Spanaus, Jan Münch, Natalia Ruetalo, Michael Schindler, Martina Morokutti-Kurz, Philipp Graf, Eva Prieschl-Grassauer, Andreas Grassauer, Ulrich Schubert

**Affiliations:** 1 Institute of Virology, Friedrich-Alexander University Erlangen-Nürnberg (FAU), 91054 Erlangen, Germany; Maria.Carolin.Froeba@fau.de (M.F.); Maximilian.Grosse@uk-erlangen.de (M.G.); Christian.Setz@uk-erlangen.de (C.S.); Pia.Rauch@uk-erlangen.de (P.R.); Janina.Auth@fau.de (J.A.); Lucas.Spanaus@extern.uk-erlangen.de (L.S.); 2 Institute of Molecular Virology, Ulm University Medical Center, 89081 Ulm, Germany; Jan.Muench@uni-ulm.de; 3 Institute for Medical Virology and Epidemiology of Viral Diseases, University Hospital Tübingen, 72076 Tübingen, Germany; Natalia.Ruetalo-Buschinger@med.uni-tuebingen.de (N.R.); Michael.Schindler@med.uni-tuebingen.de (M.S.); 4 Marinomed Biotech AG, A-2100 Korneuburg, Austria; Martina.Morokutti-Kurz@marinomed.com (M.M.-K.); Philipp.Graf@marinomed.com (P.G.); Eva.Prieschl@marinomed.com (E.P.-G.); Andreas.Grassauer@marinomed.com (A.G.)

**Keywords:** COVID-19, SARS-CoV-2, coronavirus, pseudotyping, iota-carrageenan, kappa-carrageenan, lambda-carrageenan, sulfated polymer, virus variants, variants of concern, carrageenan types

## Abstract

The COVID-19 pandemic continues to spread around the world and remains a major public health threat. Vaccine inefficiency, vaccination breakthroughs and lack of supply, especially in developing countries, as well as the fact that a non-negligible part of the population either refuse vaccination or cannot be vaccinated due to age, pre-existing illness or non-response to existing vaccines intensify this issue. This might also contribute to the emergence of new variants, being more efficiently transmitted, more virulent and more capable of escaping naturally acquired and vaccine-induced immunity. Hence, the need of effective and viable prevention options to reduce viral transmission is of outmost importance. In this study, we investigated the antiviral effect of iota-, lambda- and kappa-carrageenan, sulfated polysaccharides extracted from red seaweed, on SARS-CoV-2 Wuhan type and the spreading variants of concern (VOCs) Alpha, Beta, Gamma and Delta. Carrageenans as part of broadly used nasal and mouth sprays as well as lozenges have the potential of first line defense to inhibit the infection and transmission of SARS-CoV-2. Here, we demonstrate by using a SARS-CoV-2 spike pseudotyped lentivirus particles (SSPL) system and patient-isolated SARS-CoV-2 VOCs to infect transgenic A549ACE2/TMPRSS2 and Calu-3 human lung cells that all three carrageenan types exert antiviral activity. Iota-carrageenan exhibits antiviral activity with comparable IC_50_ values against the SARS-CoV-2 Wuhan type and the VOCs. Altogether, these results indicate that iota-carrageenan might be effective for prophylaxis and treatment of SARS-CoV-2 infections independent of the present and potentially future variants.

## 1. Introduction

By now, the COVID-19 pandemic caused by the emergence of the Severe Acute Respiratory Syndrome Coronavirus type-2 (SARS-CoV-2) has resulted in around 256 million global cases and 5.1 million global deaths [1]. While vaccination campaigns are ongoing, the emergence and spread of SARS-CoV-2 variants is becoming a major threat to public health. These “Variants of Concern” (VOC) are the result of viral evolution and have the potential to evade vaccine- or infection-induced antiviral immune responses [2,3].

SARS-CoV-2 belongs to the genus Betacoronaviruses and is closely related to SARS-CoV, which caused an outbreak of atypical pneumonia in 2002–2003 [4]. Both viruses bind to human angiotensin-converting enzyme 2 receptor (hACE2) via their heavily glycosylated spike protein [5,6,7]. Upon binding, SARS-CoV-2 can enter the cell either via membrane fusion after cleavage of the spike glycoprotein by the protease ACE2 [8] or via endocytosis facilitated by proteases, such as cathepsin L [9,10]. Recently, different SARS-CoV-2 variants, that harbor amongst other alterations mutations in the receptor-binding domain (RBD) of the spike glycoprotein [11,12,13,14], have emerged worldwide and are spreading rapidly. 

VOCs include SARS-CoV-2 Alpha [15] (also referred to as B.1.1.7 [16]), SARS-CoV-2 Beta [17] (also referred to as B.1.351 [16]), SARS-CoV-2 Gamma [18] (also referred to as P.1 [16]), as well as SARS-CoV-2 Delta [19] (also referred to as B.1.617.2 [16]). These VOCs were suggested to show higher transmissibility and infectivity [11,12,13,14,15,16,17,18,19,20,21,22,23,24], causing drastically rising numbers of COVID-19 cases worldwide. In the light of this ongoing trend, the development of broadly effective prophylactic and therapeutic countermeasures remains of the utmost importance.

There are currently no specific antiviral treatment options apart from monoclonal antibodies for high-risk patients, which need to be administered at an early time point of infection [25]. Moreover, in the light of currently spreading VOCs, the efficacy of current vaccines against mutated virus strains still needs to be evaluated conclusively. Furthermore, herd immunity might be difficult to achieve, as the vaccines do not confer sterile immunity [26]. 

Together, this highlights the unmet urgent need to develop prophylactically as well as safe therapeutic agents, which should be widely available and broadly acting against different viral strains of SARS-CoV-2. Considering the time- and cost-consuming path for the development of new therapeutics, particularly of specific monoclonal antibodies, the evaluation of existing drugs as well as natural substances for their antiviral activity against SARS-CoV-2 represent a fast and promising alternative.

Respiratory viruses enter the body mainly via the nasopharyngeal cavity. In addition to the nasal epithelium, they infect also other parts of the respiratory tract, such as the pharyngeal mucosa. Virions are transmitted from person to person by respiratory droplets, aerosols or indirectly via contaminated surfaces. A reduction of viral load at the site of infection, thereby, limits the transmission between individuals and their own lower respiratory tract [27,28,29,30]. In the past, natural substances have been highlighted repeatedly for their antiviral potential against a variety of viruses. 

Since the outbreak of the current SARS-CoV-2 pandemic, several natural substances were tested for their potential effects against SARS-CoV-2 [31,32,33]. Among them carrageenan, which is a high molecular weight sulfated polymer derived from red seaweed (Rhodophyceae) that has been extensively used in the food, cosmetic and pharmaceutical industries and is generally recognized as safe by the FDA (GRAS). Three main forms of carrageenans are commercially used: iota, kappa and lambda. They differ from each other in the degree of sulfation, solubility and gelling properties [34].

The antiviral activity of iota-carrageenan is well-established and has been demonstrated for a variety of respiratory viruses [35,36,37]. Recently, it has been shown that iota-carrageenan inhibits SARS-CoV-2 replication in various cell lines [38] and in primary differentiated human airway epithelial cultures [39]. For lambda-carrageenan an antiviral activity in Vero E6 cells against SARS-CoV-2 was reported [40]. 

Moreover, treatment of respiratory viral infection with iota-carrageenan nasal spray has already been shown to be safe and effective in five randomized, double blinded, placebo-controlled clinical trials with more than 600 children and adults suffering from respiratory viral infection. Therapeutic application of an iota-carrageenan nasal spray reduced the viral load, which also manifested clinically by reducing the severity and duration of symptoms as well as the number of relapses in the verum group [41,42,43,44,45].

Very recently, it was shown in a multicenter, randomized, double-blinded, placebo-controlled clinical study, that an iota-carrageenan containing nasal spray exhibits prophylactic efficacy in preventing SARS-CoV-2 infection in healthcare workers caring for patients with COVID-19 disease with a relative risk reduction of 79.8% [46]. An earlier trial investigating a nasal spray containing Ivermectin and iota-carrageenan showed reduction in COVID-19 as well as of disease severity [47]. Furthermore, there are clinical trials on COVID-19 cases running in the UK [48] and in Austria [49,50] investigating the prophylactic and therapeutic effect of iota-carrageenan as inhalant.

We previously published that iota-carrageenan exhibits antiviral activity against the SARS-CoV-2 Wuhan type in Vero B4 cells [38]. In our current work, we show for the first time that carrageenans exhibits antiviral activity not only against the SARS-CoV-2 Wuhan type but also the VOCs Alpha, Beta, Gamma and Delta with comparable IC_50_ values. This effect was shown for all carrageenan types with iota being the most effective, occurring in human cell lines and in the SSPL system, as shown for SARS-CoV-2 Wuhan type, and the VOCs Alpha, Beta and Gamma. 

The IC_50_ values varied depending on the form of infection and cell lines used, ranging from 1.4–5 µg/mL iota-carrageenan in the SSPL particles system to 2.1–10.3 µg/mL in A549-ACE2/TMPRSS2 cells, and 0.04–0.15 µg/mL in Calu-3 human lung cells infected with the SARS-CoV-2 variants. Thereby, iota-carrageenan displayed an at least 10-fold higher efficacy when compared to lambda- and kappa-carrageenan.

## 2. Results

### 2.1. Comparison of Iota-Carrageenan with Other Sulfated and Non-Sulfated Polymers against SARS-CoV-2 VOCs

It was shown previously that, in addition to iota-carrageenan, kappa- and lambda-carrageenan also exhibit antiviral activity against the SARS-CoV-2 Wuhan type [38,39,40]. The polysaccharides differ in their location and number of sulfate moieties on the hexose scaffold sceleton and thus contain one (kappa), two (iota) or three (lambda) negatively charged sulfate ester groups per disaccharide repeating unit [51,52]. Carrageenan homopolymers do usually not occur isolated in nature. Therefore, many of the available preparations contain relevant amounts of the other polymers. For this study, defined preparations of purified carrageenans were used, for which the quality was finally controlled by ^1^H-NMR spectroscopy as published before [38].

We first determined whether iota and the other sulfated carrageenaan types kappa and lambda block the infection of cells with lentiviral particles pseudotyped with SARS-CoV-2 Spike protein from the SARS-CoV-2 VOCs Alpha, Beta, Gamma and Delta with the same efficacy as for the Wuhan type. Therefore, ACE2-HEK293 cells were infected with the respective SSPL particles and spike driven infection was measured as described previously [38]. As control, polymers without sulpate groups, carboxymethylcellulose (CMC) and hydroxypropylmethylcellulose (HPMC) were used, as it was shown previously that these polymers have no antiviral activity against SARS-CoV-2 [38].

**Figure 1:**
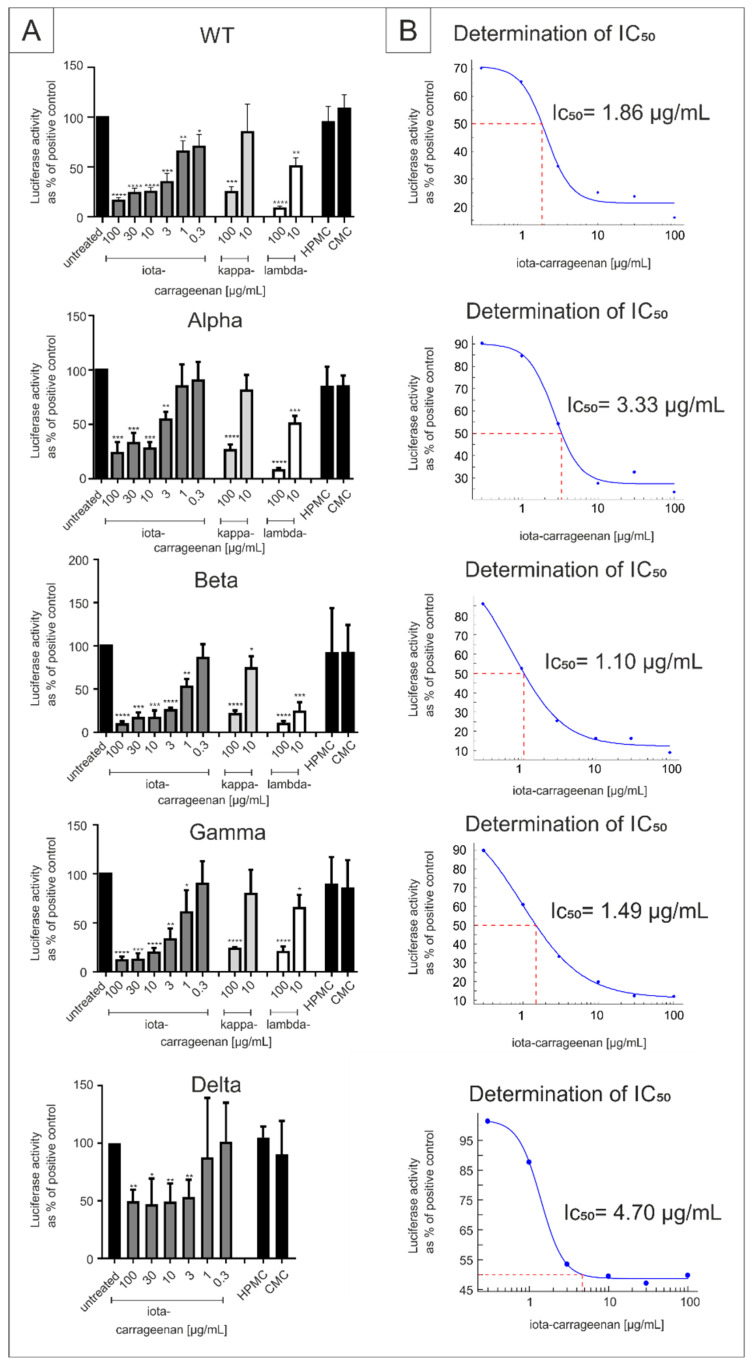
Effect of iota-, kappa- and lambda-carrageenan on SARS-CoV-2 spike driven entry. (**A**) Luciferase activity following infection of ACE2-HEK293 with SSPL particles normalized to untreated infected cells which served as positive control (100% infection control; *y*-axis). 100, 30, 10, 3, 1, 0.3 µg/mL iota-carrageenan, 100 and 10 µg/mL kappa- and lambda-carrageenan, and 100 µg/mL non-sulfated polymers (hydroxypropylmethylcellulose (HPMC), carboxymethylcellulose (CMC)) were incubated with the SSPL for 30 min before infection. The efficiency of infection in cell lysates was determined by measuring the luciferase activity 48 h post infection. (**B**) Determination of IC50 values using Excel XLfit version 5.5.0.5. The data represent means of quadruplicates ± standard deviation (* *p* < 0.5, ** *p* < 0.01, *** *p* < 0.001, **** *p* < 0.0001 using a one sample *t* test, where each value is compared to the untreated control). Abbr.: iota-carrageenan, kappa-carrageenan, lambda-carrageenan, CMC and HPMC.

Iota-carrageenan inhibited viral infection with Wuhan type pseudotyped lentivirus with an IC_50_ of 1,86 (0.30–3,43) and was similarly active against the SARS-CoV-2 Alpha, Beta, Gamma and Delta variant, with IC_50_ values of 3.33, 1.1, 1.49 and 4.70 µg/mL (Figure 1). Kappa- and lambda-carrageenan also showed some antiviral activity when used at 100 µg/mL but were hardly active at 10 µg/mL (Figure 1). As to be expected, CMC and HPMC were inactive in this assay (Figure 1).

### 2.2. Iota-Carrageenan Exhibits Comparable Antiviral Activity against Different Variants of SARS-CoV-2

**Figure 2:**
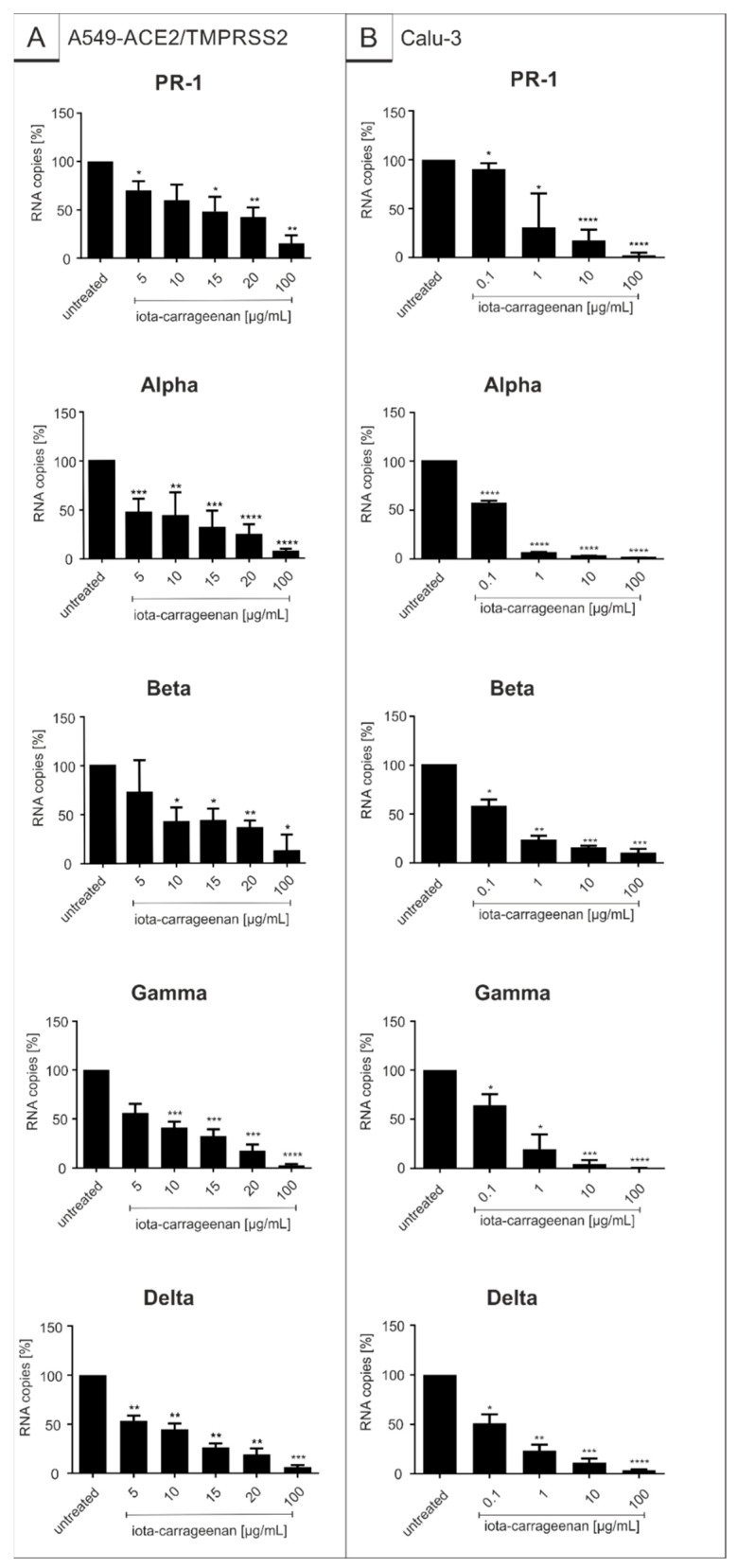
Iota-carrageenan inhibits replication of SARS-CoV-2_PR-1_ and the VOCs Alpha, Beta, Gamma and Delta with comparable efficacy. A549-ACE2/TMPRSS2 (**A**) and Calu-3 cells (**B**) were infected with either SARS-CoV-2_PR-1_, Alpha, Beta, Gamma or Delta at a MOI of 2 × 10^−2^. At 1 h after infection and removal of input virus, cells were treated with indicated concentrations of iota-carrageenan. Cell culture supernatants were harvested at 3 days post infection (dpi). The virions were purified and analyzed by q-RT-PCR. Data represent means of 6 (**A**—Alpha), 4 (**B**—PR-1) or 3 (**A**—PR-1, **A**—Beta, **A**—Gamma, **A**—Delta, **B**—Alpha, **B**—Beta, **B**—Gamma, and **B**—Delta) independent experiments ± standard deviation (* *p* < 0.5, ** *p* < 0.01, *** *p* < 0.001, and **** *p* < 0.0001) using a one sample *t* test, where each value is compared to the untreated control. The absolute values of conducted qRT-PCR analysis are included in Supplementary Table S1.

In order to determine whether the results obtained by SSPL also apply to replication competent SARS-CoV-2, transgenic A549-ACE2/TMPRSS2 cells as well as Calu-3 human lung cells representing the most extensively studied surrogate lung cell infection model expressing ACE2 and TMPRSS2 endogenously [53], were infected with the Wuhan type isolate SARS-CoV-2_PR−1_ and the respective VOCs (Figure 2). One hour post infection, different concentrations of iota-carrageenan were added to the cells. Three days post infection (dpi) cell culture supernatants were harvested, and virus production was analyzed by quantitative RT-PCR (Figure 2). The absolute values of the conducted qRT-PCR analysis are included in Supplementary Table S1.

As with SSPL, treatment with iota-carrageenan led to a strong reduction of virus replication that occurred with comparable efficacy for SARS-CoV-2_PR-1_ and the respective VOCs in the investigated cell lines. At a low concentration of 10 µg/mL, iota-carrageenan almost completely blocked the production of progeny virions. In comparison to the SSPL system and A549-ACE2/TMPRSS2 cells, treatment of Calu-3 cells with iota-carrageenan exhibits the strongest reduction of viral replication with comparable IC_50_ values for SARS-CoV-2_PR-1_ and the VOCs (0.12–1.66 µg/mL in Calu-3 cells vs. 1.1–4.7 µg/mL in the SSPL system and 5.25 µg/mL–20.75 µg/mL in A549-ACE2/TMPRSS2 cells) (Figure 1 and Figure 2, Table 1).

Previous studies revealed that in addition to iota-carrageenan, the structural related carrageenan types lambda and kappa exhibit antiviral activity against SARS-CoV-2 in vitro [38,39,40,41,42,43,44,45,46,47,48,49,50,51,52,53,54].

**Figure 3:**
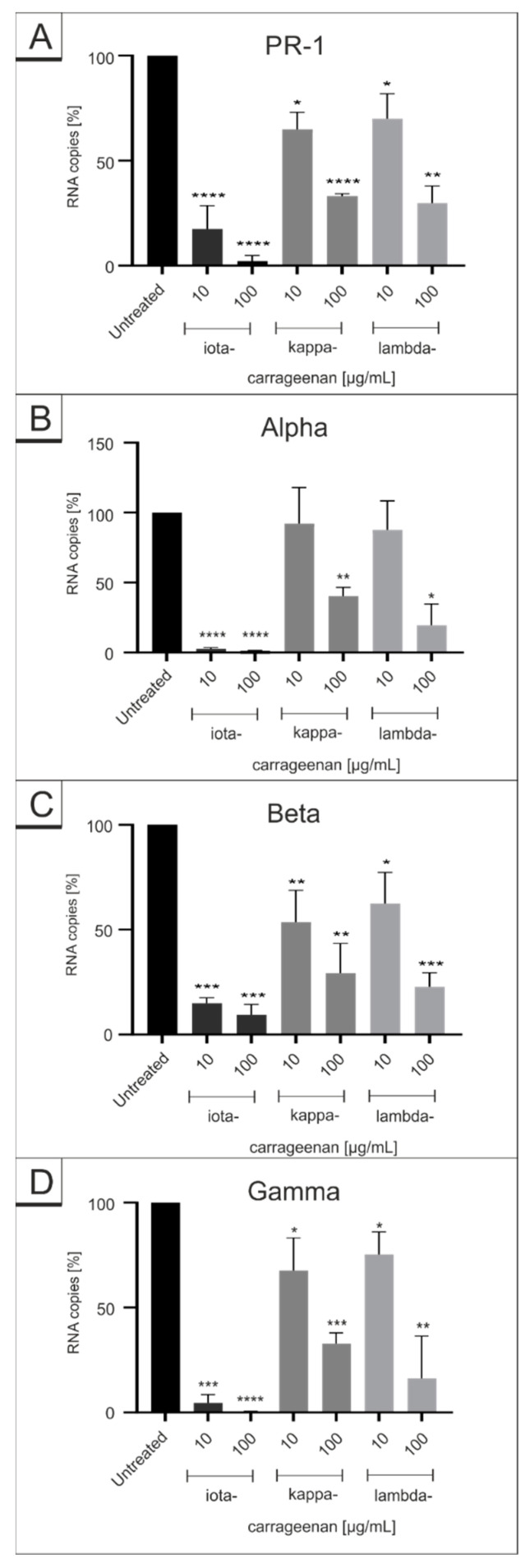
Comparison of the influence of iota-, lambda- and kappa-carrageenan on the replication of SARS-CoV-2 wt and its VOCs. Calu-3 were infected with SARS-CoV-2_PR-1_, Alpha, Beta or Gamma at a MOI of 2 × 10^−2^. At 1 h post infection, input virus was removed, and cells were treated with the indicated concentrations of iota-, kappa- and lambda-carrageenan. Cell culture supernatants were harvested at 3 days post infection (dpi). The virions were purified and analyzed by q-RT-PCR. Data represent means of 6 (**A** iota-carrageenan), 4 (**C**+**D** kappa- and lambda-carrageenan) and 3 (**A**+**B** kappa- and lambda-carrageenan, **B**+**C**+**D** iota-carrageenan) independent experiments ± standard deviation (* *p* < 0.5, ** *p* < 0.01, *** *p* < 0.001, and **** *p* < 0.0001) using a one sample *t* test, where each value is compared to the untreated control. The absolute values of conducted qRT-PCR analysis are included in Supplementary Table S2.

Thus, we investigated whether different carrageenans exert similar antiviral effects against the Wuhan type SARS-CoV-2_PR-1_ and its variants Alpha, Beta and Gamma following infection of Calu-3 cells (Figure 3, absolute values of conducted qRT-PCR are included in Supplementary Table S2). Although all carrageenan types exhibit antiviral activity against SARS-CoV-2_PR−1_ and the VOCs, iota-carrageenan was the most effective (Figure 3). Thereby, the IC_50_ values of iota-carrageenan were ~1 log-stage lower than that of kappa- and lambda-carrageenan. However, as with iota-carrageenan, the IC_50_ values of kappa- and lambda-carrrageenan between SARS-CoV-2_PR-1_ and the VOCs were again in a similar range indicating that all types of carrageenans acting on SARS-CoV-2 with the same mechanism. The absolute values of the conducted qRT-PCR analysis are included in Supplementary Table S2.

**Figure 4:**
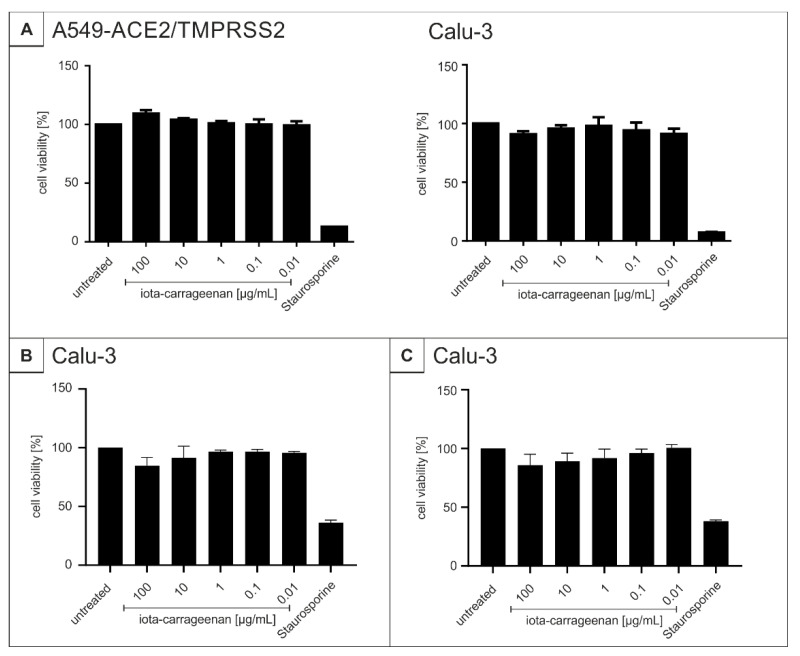
Influence of iota- (**A**), kappa- (**B**) and lambda-carrageenan (**C**) on the cell viability of A549-ACE2/TMPRSS2 cells and Calu-3 cells. Following treatment with different concentrations of carrageenan (iota-carrageenan concentrations are indicated at the *x*-axis) for three days, the influence on cell viability was measured by water-soluble tetrazolium salt (WST)-1 assay. Bars represent means of three independent experiments ± SD. Staurosporine (1 µM) was used as a positive control.

To control for potential unspecific effects of carrageenan treatment on cell viability, water-soluble tetrazolium salt (WST)-1 assays were performed in uninfected A549-ACE2/TMPRSS2 or Calu-3 cells under otherwise identical conditions as for infection experiments. The results summarized in Figure 4 demonstrate that treatment with all carrageenan types at concentrations up to 100 µg/mL, which more than effectively suppress SARS-CoV-2 replication in all tested settings, exhibit no impact on cell viability in both cell types (Figure 4). Staurosporine was used as a positive control at a concentration of 1 µM.

In summary, and in consistency with previous reports [38] a relative 3-fold therapeutic window for iota-carrageenan can be expected in those analyzed cell types.

## 3. Discussion

Since the beginning of the outbreak in December 2019, the causative virus of the COVID-19 pandemic exerted a dramatic health and socioeconomic crisis worldwide. It can be assumed that, as before for SARS-CoV and MERS-CoV, in the future, coronaviruses could spread from animals to humans via zoonotic transmission, potentially causing pandemic threats. This clearly necessitates a general need for pandemic preparedness. Regarding SARS-CoV-2, worldwide vaccination programs remain a challenge as inefficiency and a lack of supply particularly in developing countries might lead to the emergence of new variants that may be more efficiently transmitted, more virulent and more capable of escaping naturally acquired and vaccine-induced immunity. 

Thus, there is a tremendous need for the development of new therapeutics that are broadly active, safe, relatively cheap and easily distributable for a wide range of patients when compared to standard antivirals. As an alternative approach to the repurposing of existing synthetic drugs, like hydroxy-chloroquine and remdesivir, which was proven ineffective [55], natural substances would have the advantages of a better toxicological profile with a larger therapeutic window, less side effects and a faster admission process in comparison to chemically engineered drugs. 

In addition, due to their general mode of action, most naturals exhibit a broad antiviral spectrum when compared, for instance, to highly specific monoclonal antibodies or small molecule inhibitors of viral factors. In the past, the beneficial effects of natural substances were shown for many diseases, including metabolic disorders or cancer [56]. Most importantly, they also proved promising against a variety of different viruses, including SARS-CoV, MERS-CoV and SARS-CoV-2 [31,32,33].

In this study, we demonstrate that the natural substance iota-carrageenan, exerts antiviral activity not only against the originally emerged SARS-CoV-2 Wuhan type but also against the VOCs Alpha, Beta, Gamma and Delta. Data from a SSPL system as well as from transgenic A549-ACE2/TMPRSS2 and human Calu-3 lung cells were comparable indicating that carrageenans exhibit a broad antiviral activity.

The antiviral effect of iota-carrageenan was shown against several respiratory viruses in vitro [35,36,37,38,39,40,41,42,43,44,45,46,47,48,49,50,51,52,53,54,55,56,57,58,59] and was also proofed in clinical trials [41,42,43,44,45,46]. Interestingly, a very recent study revealed a synergistic antiviral activity using a combination of carrageenan with another natural substance, the lectin Griffithsin, when tested against SARS-CoV-2 pseudoviruses [54].

Hemilä et al. performed an independent meta-analysis with publicly available data from clinical studies with an iota-carrageenan containing nasal spray [60]. The authors attest the quality of the available clinical data as well as the broad effectiveness against influenza viruses and endemic coronaviruses [60]. They further conclude that carrageenan may also inhibit the new coronavirus SARS-CoV-2, an assumption that was confirmed by a recent clinical study showing that an iota-carrageenan containing nasal spray give significant protection when used as COVID-19 prophylaxis in health care workers managing patients with COVID-19 disease [46]. 

Furthermore, another recently published clinical study underlined this assumption as it demonstrated that sucking iota-carrageenan containing Lozenges inhibit viral replication of SARS-CoV-2 ex vivo [61]. Moreover, there are regional different recommendations for the use of iota-carrageenan in prevention of COVID-19, as for example by the German Society of Hospital Hygiene and by Austrian society [62,63].

Nasal sprays, throat sprays and lozenges containing carrageenans are approved common cold prevention options and have been launched in more than 20 countries [64]. The most commonly used carrageenan in these products is iota-carrageenan. To test whether the types of carrageenan have different antiviral properties, we compared iota-, kappa- and lambda-carrageenan in various infection experiments. Although all carrageenan types showed antiviral effects against SARS-CoV-2 Wuhan type and its VOCs Alpha, Beta and Gamma, iota-carrageenan clearly led to the strongest reduction with IC_50_ values that are ~1 log-stage lower than that for lambda- or kappa-carrageenan. 

These results are in concert with other studies were it was shown that iota-carrageenan exhibits the most potent reduction of human rhinovirus infection and hepatitis A virus replication when compared to kappa- or lambda-carrageenan [35,36,37,38,39,40,41,42,43,44,45,46,47,48,49,50,51,52,53,54,55,56,57,58,59,60,61,62,63,64,65]. The molecular basis for the observed difference, however, remains widely unclear. Interpretation of data is hampered by the finding, that even purchased preparations of kappa- and lambda-carrageenan contained 16 % and 27.3 % iota-carrageenan, respectively [38], thus, indicating that the observed antiviral activity of kappa- and lambda-carrageenan is rather due to the presence of iota-carrageenan than an effect of the respective polymers themselves.

There are some differences between the IC_50_ values of iota-carrageenan obtained for the SSPL and SARS-CoV-2 systems (Table 1). Pseudo-typed virus-like particles versus replication competent viruses react different to carrageenans, as also non-human Vero as well as human lung cells exhibit differences. In addition, A549 cells are transgenic for ACE2/TMPRSS2, whereas Calu-3 express ACE2 and TMPRSS2 endogenously [31,32,33,34,35,36,37,38,39,40,41,42,43,44,45,46,47,48,49,50,51,52,53,54,55,56,57,58,59,60,61,62,63,64,65,66]. However, there was a clear antiviral activity with comparable IC_50_ values against the SARS-CoV-2 Wuhan type and the VOCs following treatment with iota-carrageenan for all systems.

Carrageenans not only have antiviral properties but also exert antitumor, immunomodulatory and coagulative activities, respectively [58,59,60,61,62,63,64,65,66,67,68,69,70,71]. In this context, it was published that kappa- and lambda-carrageenans exhibit both anticancer and immunostimulatory effects [58,59,60,61,62,63,64,65,66,67,68,69,70]. In contrast, such immunomodulating and antitumoral effects have not been described yet for iota-carrageenan, supporting a broad application as an otherwise biologically inert substance. Furthermore, iota-carrageenan has been approved as food safe by the EFSA [72] and by the FDA [73] for a quantum satis of 75 mg/kg b.w. per day which would translate into 10 L of plum pudding [72]. 

The sulfated polysaccharide does not enter the human cells as it is degraded before [73]. In concert with this, a non-clinical study analyzing iota-carrageenan showed no local intolerance or toxicity when iota-carrageenan was applied intranasally or by inhalation [74]. In addition, no immunogenicity or immunotoxicity were observed as there was no stimulation of a panel of pro-inflammatory cytokines by iota-carrageenan detectable [74]. 

In agreement with this, our data showed no toxic effect on A549-ACE2/TMPRSS2+ and Calu-3 cells when treated with different concentrations of iota-carrageenan up to 100 µg/mL. It is intriguing to hypothesize that iota-carrageenan, which shows the best antiviral activity in comparison with the other types of carrageenan, is biologically inert and, thus, can be used against SARS-CoV-2 and variants in the ongoing pandemic without any concern in the view of adverse effects.

Various carrageenans and other sulpated polysaccharides, like heparin, dextran sulfate and pentosan sulfate, exert antiviral activity against enveloped viruses [75,76,77,78,79]. In order to interact with their specific host cell receptors [38], it is hypothesized that viruses utilize their positive electrical charge to reach the negatively charged cell surface [80]. Polyanionic molecules, such as iota-carrageenan may present a way of trapping the virus due to their affinity for basic viral surfaces [81]. As iota-carrageenan non-specifically envelops viruses, thereby, preventing interaction between virus and cellular surface, the development of resistance due to the occurrence of escape mutants is unlikely. 

This current knowledge is backed by a series of experiments showing that direct interaction between virus and carrageenan is needed to efficiently inhibit infection of cells [35,36,37,38,39,40,41,42,43,44,45,46,47,48,49,50,51,52,53,54,55,56,57,58,59,60,61,62,63,64,65,66,67,68,69,70,71,72,73,74,75,76,77,78,79,80,81,82]. The activity of carrageenan is based on its ability to neutralize virus particles when they first enter the nasal cavity, and additionally when newly synthesized virus particles are released from infected cells. The long, negatively charged iota-carrageenan molecules attract and trap these newly released positively charged viruses and sterically hinder them from binding to their host cells, thereby, inhibiting the infection of adjacent epithelial cells. Finally, iota-carrageenan together with the trapped viruses will then be eliminated by mucociliary clearance [80].

The lack of any pharmacological, immunological or toxicological activity of large polyanionic molecules such as iota-carrageenan and their lack of absorption or metabolism makes them a safe biologically inert antiviral that can be applied topically, e.g., as lozenges or nasal spray. Since iota-carrageenan has similar IC_50_ values following infection with different VOCs, a covalent selective interaction of carrageenan with the spike protein of SARS-CoV-2 appears rather unlikely, while electrostatic encasement of virions mediated by the sulpated polysaccharide supports prophylactically application of this naturally occurring substance against SARS-CoV-2 regardless of newly emerging variants.

## 4. Materials and Methods

### 4.1. Inhibitors

Iota-, kappa- and lambda-carrageenan were purchased from Dupont former FMC Biopolymers (both Philadelphia, PA, USA). Fucoidan from Undaria pinnatifolia and Fucus vesiculosis were from Marinova (Marinova Pty Ltd., Cambridge TAS, Australia), Carboxymethylcelluslose (CMC) was from Mare Austria GmbH, Niklasdorf, Austria Hydroxypropylmethylcellulose (HPMC) from Fagron (Fagron BV, Rotterdam, The Netherlands). The dry polymer powders were dissolved in cell culture water (B Braun Melsungen AG, Melsungen, Germany) to a final concentration of 2.4 mg/mL containing 0.5% NaCl (Merck KGA, Darmstadt, Germany). This stock solution was sterile filtered through a 0.22 mm filter (Sarstedt, Nümbrecht, Germany) and stored at 4 °C until use.

### 4.2. SARS-CoV-2 Spike Pseudotyped Lentivirus (SSPL)

Pseudotyped particles were obtained from BPS Bioscience, San Diego, CA, USA. Catalog: #79942 (wildtype), #78112 (Alpha), #78142 (Beta), #78144-2 (Gamma) and #78205 (Delta). The pseudovirions contain SARS-CoV-2 Spike protein (Genbank Accession #QHD43416.1) and the firefly luciferase gene driven by a CMV promoter. Therefore, the spike-mediated cell entry can be measured via luciferase reporter activity. The SARS-CoV-2 Spike pseudotyped lentivirus was designed to measure the activity of neutralizing antibody against SARS-CoV-2 in a BSL2 facility.

### 4.3. Neutralization Test

The pseudotyped virus was used according to the manufacturer’s instructions: Approximately 7500 ACE2-HEK293 cells/well were infected with 750 infectious particles (MOI = 0.1) of SARS-CoV-2 Spike pseudotyped lentivirus (Luc reporter). The SSPL were incubated with buffer (controls) or the test substances for 30 min before infection (designated as concentration “virus contact”). For infection, five volumes of cell culture medium (MEM (Merck KGA, Germany) containing 10% FCS (Merck KGA, Germany), 4 mM glutamine (Merck KGA, Germany), 1 mM sodium pyruvate (Merck KGA, Germany), and 1% penicillin/streptomycin (Merck KGA, Germany) were added, resulting in 1:6 dilution of the initial concentration of the test substance. 

At 24 h after infection, the medium was changed to fresh cell culture medium. At 48 h after infection, plates were lysed by freeze/thaw before luciferase reagent (Bright Glow, Promega, Madison, WI, USA) was added to cells to measure the luciferase activity using a BMG Fluostar Microplate reader. Mock-infected cells and infected, mock-treated (0.5% NaCl) cells served as positive and negative control. Luciferase data were routinely corrected with metabolic data (Alamar blue) derived from a parallel plate with an identical setup.

### 4.4. Viruses

The “Wuhan type” virus SARS-CoV-2_PR−1_, isolated from a 61 year old patient, was amplified in Vero B4 cells as described in [31]. The virus strains SARS-CoV-2 Alpha, Beta, Gamma and Delta were obtained from Michael Schindler (University Hospital, Tübingen). The SARS-CoV-2 Alpha variant (210416_UKv) was generated as described in [33]. SARS-CoV-2 Beta was generated as described in [83]. The Gamma (210504_BRv) and the Delta variant (210601_INv) were isolated from throat swabs collected in May 2021 at the Institute for Medical Virology and Epidemiology of Viral Diseases, University Hospital Tübingen, from PCR-positive patients. 

In total, 40–200 µL of patient material were diluted in medium and used to directly inoculate 150,000 Caco-2 cells in six-well plates. 48–72 h post-infection, supernatants were collected, centrifuged, and stored at −80 °C. After two consecutive passages, RNA samples from the supernatant were prepared, and next generation sequencing (NGS) confirmed that the clinical isolates belong to the lineage P.1 and B.1.617.2., respectively. Viral titers were determined by an endpoint titration assay. For the generation of new virus stock, virus containing cell culture supernatant was harvested 72 h post infection (hpi) and passed through a 0.45 μm pore-size filter. Virus stocks were stored at −80 °C until further usage.

### 4.5. Infection Experiments

For infection experiments, cells were inoculated with SARS-CoV-2_PR-1_ or the VOCs Alpha, Beta, Gamma and Delta (multiplicity of infection (MOI): 2 × 10^−2^) for 1 h, washed and further treated with interventions. 72 hpi, virus-containing cell culture supernatants were incubated for 10 min at 95 °C and finally used for qRT-PCR analysis. For titer determination of SARS-CoV-2 virus stocks, A549-ACE2/TMPRSS2 and Calu-3 cells were infected with serial dilutions of the virus stock over 72 h. Afterwards cells were fixed (4% PFA), permeabilized (0.5% Triton/PBS), blocked (1% BSA/PBS-T) and finally stained with a SARS-CoV-2 NP antibody (Biozol). The endpoint of virus infection was analyzed via fluorescence microscopy and viral titer was calculated by the method of Reed and Muench [84].

### 4.6. Cell Culture

Calu-3 cells were maintained in Minimal Essential Medium (MEM) containing 20% (*v*/*v*) inactivated FCS, 1 mM l-glutamine, 100 U/mL penicillin, and 100 μg/mL streptomycin and 1 mM sodium pyruvate. A549-ACE2/TMPRSS2 cells were maintained in RPMI 1640 Medium containing 10% (*v*/*v*) inactivated fetal calf serum (FCS), 1 mM l-glutamine, 100 U/mL penicillin, and 100 μg/mL streptomycin, 0.075% sodium bicarbonate and 1 µg/mL puromycin and blasticidin. A549-cells expressing ACE2 and TMPRSS2 were generated by retroviral transduction as described in [31] and cultivated in RPMI 1640 medium containing 10% (*v*/*v*) inactivated fetal calf serum (FCS), 2 mM l-glutamine, 100 U/mL penicillin, 100 μg/mL streptomycin and 100 µg/mL blastomycin.

### 4.7. Assessment of Cell Viability

The viability of uninfected and treated cells was assessed by the water-soluble tetrazolium salt (WST)-1 assay (Roche) and by neutral red assay according to the manufacturer’s instructions. Cells were treated for 72 h with various inhibitors according to the protocols of the infection experiments.

### 4.8. Determination of the Amount of Viral RNA Copies from Released Viruses by qRT-PCR

Virus was quantified by real-time PCR AgPath-ID One-Step RT-PCR Kit from Ambion (Cat: 4387424) software v2.3 (applied Bioscience). PCR primers were used according to [85]: RdRp_fwd: 5′-GTG-ARA-TGG-TCA-TGT-GTG-GCG-G-3′ and RdRp_rev 5′-CAR-ATG-TTA-AAS-ACA-CTA-TTA-GCA-TA-C-3′. Probe was 5′-CAG-GTG-GAA-/ZEN/CCT-CAT-CAG-GAG-ATG-C-3′ (Label: FAM/IBFQ Iowa Black FQ). As a positive control, a specific target for E and RdRp gen of SARS-CoV2 was used and made by Integrated DNA Technologies. 

Control: 5’-TAA-TAC-GAC-TCA-CTA-TAG-GGT-ATT-GAG-TGA-AAT-GGT-CAT-GTG-TGG-CGG-TTC-ACT-ATA-TGT-TAA-ACC-AGG-TGG-AAC-CTC-ATC-AGG-AGA-TGC-CAC-AAC-TGC-TTA-TGC-TAA-TAG-TGT-TTT-TAA-CAT-TTG-GAA-GAG-ACA-GGT-ACG-TTA-ATA-GTT-AAT-AGC-GTA-CTT-CTT-TTT-CTT-GCT-TTC-GTG-GTA-TTC-TTG-CTA-GTT-ACA-CTA-GCC-ATC-CTT-ACT-GCG-CTT-CGA-TTG-TGT-GCG-TAC-TGC-TGC-AAT-ATT-GTT-3’.

### 4.9. NMR Analysis of Kappa- and Lambda-Carrageenan

We sent 10 mg kappa and lambda-carrageenan samples to Spectral Services, Köln, for NMR measurements. In brief, 10 mg substance was dissolved in 1 mL D2O containing 3-(trimethylsilyl) propionic acid-d4 sodium salt (0.01% as standard). Measurements for 1H spectra were done with an Avance III HD 500 MHz NMR spectrometer (Bruker, Billarica, MA, USA).

## Figures and Tables

**Table 1:** IC_50_ of Iota-carrageenan against SARS-CoV-2 wt and its VOCs in a SSPL system, A549-ACE2/TMPRSS2 cells and Calu-3 cells. The 95% confidence interval (CI) is given in brackets.

Virus Strain	Iota-Carrageenan IC_50_ (CI) [µg/mL]		
	SSPL	A549-ACE2/TMPRSS2	Calu-3
SARS-CoV-2_PR-1_	1.86 (0.3–3.9)	20.75 (7.2–34.30)	1.66 (−30.01–32.33)
Alpha	3.33 (1.45–5.21)	5.25 (2.13–8.37)	0.12 (0.09–0.15)
Beta	1.10 (0.56–1.59)	10.32 (5.82–14.82)	0.15 (−0.14–0.44)
Gamma	1.49 (1.22–1.75)	7.54 (−10.2–25.3)	0.76 (−12.21–13.73)
Delta	4.70 (0.70–8.69)	7.56 (−4.4–19.5)	0.47 (12.04–12.04)

## Data Availability

Data are included in the article.

## Supplementary Materials

The following are available online at https://www.mdpi.com/article/10.3390/ijms222413202/s1.
