# Supplementary material for: Iota-Carrageenan Inhibits Replication of SARS-CoV-2 and the Respective Variants of Concern Alpha, Beta, Gamma and Delta
Source: Int J Mol Sci. 2021 Dec 8;22(24):13202. doi: 10.3390/ijms222413202 (PMC8709357; doi:10.3390/ijms222413202)
Supplement: Supplementary file 1 [file ijms-22-13202-s001.zip › ijms-1446945-supplementary.pdf]

Table S1: Absolute values of RNA copies obtained by qRT-PCR for figure 2. Data represent means of 6 (A549-ACE2/TMPRSS2), 4 (Calu-3 - PR-1) or 3 (rest of data sets) independent experiments.

**A549-ACE2/TMPRSS2**					
	**PR-1**	**Alpha**	**Beta**	**Gamma**	**Delta**
	viral RNA copies [$\times 10^3$]				
**untreated**	109	795	184	326	33
**5 µg/mL**	73	369	125	168	17
**10 µg/mL**	58	359	73	122	14
**15 µg/mL**	46	244	77	95	8
**20 µg/mL**	42	176	68	47	6
**100 µg/mL**	13	54	20	5	2

**Calu-3**					
	**PR-1**	**Alpha**	**Beta**	**Gamma**	**Delta**
	viral RNA copies [$\times 10^3$]				
**untreated**	5453	2932	1984	1537	112
**0.1 µg/mL**	4811	1655	1137	1032	57
**1 µg/mL**	1694	175	453	354	27
**10 µg/mL**	898	81	297	84	13
**100 µg/mL**	23	35	188	7	4

Table S2: Absolute values of RNA copies obtained by qRT-PCR for figure 3. Data represent means of 4 (Beta and Gamma) and 3 (PR-1 and Alpha) independent experiments.

		PR-1	Alpha	Beta	Gamma
		viral RNA copies [$\times 10^3$]			
	untreated	2802	5100	635	4724
**kappa-carrageenan**	10 µg/mL	1938	4483	320	3600
	100 µg/mL	941	1999	122	1518
**lambda-carrageenan**	10 µg/mL	2044	4435	380	3312
	100 µg/mL	964	1535	124	1801
